# Ionic liquid-supported magnetite nanoparticles as electrode modifier materials for estrogens sensing

**DOI:** 10.1038/s41598-020-58931-6

**Published:** 2020-02-06

**Authors:** Fernanda Moreira, Edson Roberto Santana, Almir Spinelli

**Affiliations:** Grupo de Estudos de Processos Eletroquímicos e Eletroanalíticos, Universidade Federal de Santa Catarina, Campus Universitário Reitor João David Ferreira Lima, Departamento de Química – CFM, 88040-900 Florianópolis, SC Brazil

**Keywords:** Chemistry, Analytical chemistry, Sensors

## Abstract

This paper reports the application of a carbon paste electrode modified with magnetite nanoparticles and the ionic liquid 1-butyl-3-methylimidazolium hexafluorophosphate in the electroanalytical determination of 17β-estradiol and estriol. These estrogens are potential endocrine disruptors and thus it is relevant the development of devices for their monitoring. Transmission electron microscopy, scanning electron microscopy and zeta potential techniques were applied to characterization of the modifier materials. In cyclic voltammetry experiments, irreversible oxidation peaks were observed for 17β-estradiol and estriol at +0.320 V and +0.400 V, respectively. The anodic currents obtained were approximately three times greater than those provided by the unmodified electrode due to the presence of magnetic nanoparticles and the ionic liquid, which improved the sensitivity of modified electrode. For the analysis, the parameters of the square-wave voltammetry (scan increment, amplitude and frequency) were optimized by Box-Behnken factorial design for each estrogen. For 17β-estradiol in B-R buffer pH 12.0, the calibration plot was linear from 0.10 to 1.0 μmol L^−1^, with a detection limit of 50.0 nmol L^−1^. For estriol in B-R buffer pH 11.0, the linear range was 1.0 to 10.0 μmol L^−1^, with a detection limit of 300.0 nmol L^−1^. The modified electrode was applied in the determination of 17β-estradiol and estriol in pharmaceutical formulations and the results were comparable to those obtained using UV/VIS spectrometry. Statistical tests were applied to evaluate the results and it was concluded that there was no significant difference regarding the precision and accuracy of the data provided by the two methods.

## Introduction

Endocrine disrupting chemicals (EDCs) are substances capable of disturbing the endocrine system even in low levels (ng L^−1^), affecting mainly human development and reproduction^1,2^. This group of chemicals currently includes more than 800 different chemical structures that have been found in air, soil, drinking water and foodstuffs of plant and animal origin, personal care products, fuels, pharmaceuticals, and natural or synthetic hormones^3^.

Estrogens are a class of compounds included in the broad group of EDCs^4^. Estrone (E1), 17β-estradiol (E2) and estriol (E3) are three natural estrogens found in human and animal and are therefore excreted by them. Consequently, these estrogens can enter the environment through discharges of domestic sewage effluents, runoff from agricultural sources and the disposal of animal waste, since they are not efficiently removed by conventional methods of water treatment^4,5^.

E2 is the major female hormone with the highest endocrine disrupting activity^6^. It is important for the regulation of the menstrual cycle and for the development of sexual characteristics^7^. A deficiency of E2 can lead to diseases such as hyperandrogenism, cancer, heart disease, osteoporosis and menopausal symptoms^6^. However, an excess of this hormone in the human body can cause disorders in the endocrine system, premature puberty in children, abnormality of growth and increased risk of ovarian and breast cancer in women^8^. Human and animal excretion has been cited as the main route of E2 contamination in the aquatic environment and humans, with the subsequent transport of this substance in sewage effluent^8^. Another reported form of contamination is as a residue in food, because of the illegal use of this estrogen in the rearing or hormonal treatment of animals^9^.

E3 is a hormone produced during pregnancy. Hence, E3 can be detected in maternal blood or urine and is used as a marker of fetal health and wellbeing^10^. E3 is widely used to prevent and treat disorders caused by hormone deficiency, heart disorders, cancer, hyperandrogenism, osteoporosis and urogenital diseases in women^11^. However, an excess of E3 can disrupt the endocrine system causing impairment to growth and reproduction^12^. In living organisms, the highest levels of E3 are found in fatty tissue, while in the environment they are found in soils, sediments and sludge^10^. Thus, the control of E2 and E3 levels in both the human body and the environment, especially in drugs, is of great importance and therefore there is a need for fast and low-cost methods for their sensing.

The interest in electroanalytical methodologies has been growing, especially for simplicity, miniaturization and portability of instruments, and the use of modified electrodes, which aimed at improving the sensitivity of the analyzes and lowering the detection and quantification limits^13,14^. Accordingly, various sensors have been developed and employed for the quantification of E2 and E3 in different samples. Ozcan and co-workers^15^ developed a carbon paste electrode (CPE) modified with cysteamine self-assembled gold nanoparticles, graphene nanoribbon and fumed silica for the determination of E2 in milk and pharmaceutical samples with a limit of detection of 7.4 nmol L^−1^. Antoniazzi and collaborators^7^ developed a copper(II) oxide modified carbon paste electrode for the determination of E2 in human urine and buttermilk samples. The calibration plot was linear from 60.0 to 800.0 nmol L^−1^ with a limit of detection of 21.0 nmol L^−1^. Exploring the versatility of electroanalytical methods, Ochiai and co-workers^16^ constructed a device based on cotton thread as a microfluidic channel for the quantification of E3 using a carbon nanotube modified screen-printed carbon electrode. The electrochemical detector was applied to the amperometric determination of E3 in pharmaceutical samples, with a detection limit of 530.0 nmol L^−1^. Donini and co-authors^17^ applied reduced graphene oxide-silver nanoparticles composite immobilized on glassy carbon electrode to the determination of E3 in fortified samples of tap water and synthetic urine. They achieved a detection limit of 21.0 nmol L^−1^ for E3.

In recent years, magnetite nanoparticles (Fe_3_O_4_ NPs) have attracted considerable interest for the modification of electrodes due to their particular attributes, such as large surface area, good biocompatibility, strong superparamagnetism, low toxicity and ease of preparation^18,19^. Another class of compounds that has attracted the attention of researchers as modifiers of electrodes are ionic liquids because of their high ionic conductivity, good chemical and thermal stability, wide electrochemical potential window and catalytic activity^20^. In our group, Moreira and co-authors^21^ optimized the conditions in a 3^2^ factorial design in the development of a Fe_3_O_4_ NPs and ionic liquid 1-butyl-3-methylimidazolium hexafluorophosphate (BMI.PF_6_) modified CPE. This electrode was employed for the electroanalytical determination of estrone (E1) in pork meat samples by square-wave voltammetry (SWV). Under optimized conditions, the calibration plot obtained for E1 showed two linear ranges: from 4.0 to 9.0 μmol L^−1^ and from 9.0 to 100.0 μmol L^−1^. The detection limit was calculated as 470.0 nmol L^−1^.

Within this context, in this paper we describe the application of a CPE modified with Fe_3_O_4_ NPs and the ionic liquid BMI.PF_6_ for the quantification of E2 and E3. For the first time, the estrogens were successfully individually determined by SWV in pharmaceutical products using the developed modified electrode. The experimental parameters were optimized for each estrogen with the purpose of obtaining the more sensitive methodology for each one. Hence, firstly, the Fe_3_O_4_ NPs-BMI.PF_6_/CPE was prepared according to the optimized conditions in a 3^2^ factorial design. Next, The SWV parameters were optimized employing a Box-Behnken factorial design^22^. As will be demonstrated, the optimization carried out using multivariate designs made the electrode sensitive to E2 and E3 at the nanomolar level.

## Experimental

### Reagents, solutions and samples

All chemicals were of analytical grade (Sigma-Aldrich) and used without further purification. According to the manufacturer, the size of Fe_3_O_4_ NPs is <50 nm. The solutions were prepared with water purified using a Milli-Q system (Millipore, USA) with a resistivity of 18.2 MΩ cm. Britton-Robinson (B-R) buffer, Ringer’s buffer and a NaOH solution were used for the studies on the supporting electrolyte composition and solution pH. Stock solutions of each supporting electrolyte were prepared at a concentration of 0.2 mol L^−1^ and their pH was adjusted to the appropriate value with 1.0 mol L^−1^ HCl or NaOH. A stock solution of E2 and E3 was prepared daily in ethanol at a concentration of 4.0 mmol L^–1^. Less concentrated solutions were prepared by dilution with ethanol. The buffers and stock solution of E2 and E3 were stored under refrigeration at 5 °C.

To prepare the samples, the pharmaceutical formulations containing E2 (tablets and gel) and E3 (tablets and cream) were purchased from a local drugstore in Florianópolis, Brazil. Samples of E2 (1.0 mg per tablet) and E3 (1.0 mg per tablet) were separately ground from a sampling of ten tablets. The obtained mass was homogenized with 80.0 mL of ethanol under ultrasonic stirring for 10 min. Then, the samples were filtered and transferred to a 100 mL volumetric flask, which was packed with ethanol^15^. For the E2 gel samples, 5.0 g of gel was weighed and solubilized in 100 mL of ethanol. According to the manufacturer, the gel contained 0.1% (w/w) of E2. For the E3 cream samples, 1.0 mg of cream was weighed and solubilized in 50.0 mL of ethanol. According to the manufacturer, the cream contained 0.1% (w/w) of E3. Suitable aliquots of the resulting solutions were transferred to the electrochemical cell containing 10.0 mL of the supporting electrolyte.

### Characterization of the materials

For the morphological characterization of the Fe_3_O_4_ NPs dispersion, the transmission electron microscopy (TEM) technique was used employing a high-resolution transmission electron microscope, model JEM-2100 (JEOL, Japan), operating at 100 kV. For the TEM analysis, 5.0 μL of the Fe_3_O_4_ NPs dispersed in BMI.PF_6_ was dripped onto a millimetric carbon-coated copper grid (300 mesh), which was then kept at room temperature for 12 h for complete drying. The mean size (diameter) of the points, using the ImageJ software was estimated by counting 150 randomly chosen particles on the images obtained. The CPE and Fe_3_O_4_ NPs-BMI.PF_6_/CPE were characterized by field emission gun scanning electron microscopy (FEG-SEM) using a JEM-2100 (JEOL, Japan). Samples of carbon paste were placed on the surface of a stub with area of 1.5 cm^2^ containing silver glue as adhesive. After drying for 10 min, the excess material was removed. The samples were stored in a vacuum desiccator at room temperature for 12 h. The zeta experiments were performed using a Zetasizer Nano ZS instrument (Malvern Instruments, UK), the Fe_3_O_4_ NPs dispersed in ultrapure water and the Fe_3_O_4_ NPs-BMI.PF_6_ (1:2 w/w) dispersion were measured at 25 °C.

### Preparation of electrodes

The Fe_3_O_4_ NPs-BMI.PF_6_/CPE was prepared according to the optimized conditions in a 3^2^ factorial design previously described^21^. A paste with a mass of 200.0 mg was prepared by mixing 133.0 mg of graphite powder and 7.0 mg of the Fe_3_O_4_ NPs for 10 min until a uniform blend was obtained. In the next step, 45.0 mg of mineral oil was added, and the mixture was macerated for 10 min. Then, 15.0 mg of BMI.PF_6_ was add, which was macerated again for 10 min to obtain the modified paste. For comparison purposes, Fe_3_O_4_ NPs/CPE, BMI.PF_6_/CPE and CPE were also prepared. For the Fe_3_O_4_ NPs/CPE, 133.0 mg of graphite powder with 7.0 mg Fe_3_O_4_ NPs were mixing for 10 min until a uniform blend was obtained. After, 60.0 mg of mineral oil was added, and the mixture was manually macerated for 20 min to obtain the Fe_3_O_4_ NPs/CPE. For the BMI.PF_6_/CPE electrode, 140.0 mg of graphite powder with 45.0 mg of mineral oil were macerated for 10 min. Then, 15.0 mg of BMI.PF_6_ was added and the mixture was macerated for 20 min. The order in which the binders (mineral oil and ionic liquid) were added to form the paste did not interfere in the modified electrode response. Finally, an unmodified CPE was prepared with 140.0 mg of graphite powder and 60.0 mg of mineral oil. The mixture was macerated for 30 min to obtain the paste. The pastes were then packed into the 1.0 mL plastic syringes. Electrical contacts were obtained using copper wires. The paste electrodes were abraded manually to ensure renewed surfaces before each measurement.

### Measurements

Cyclic voltammetry (CV) and square-wave voltammetry (SWV) measurements were carried out with a portable potentiostat EmStat2 (Palm Instruments BV, The Netherlands), coupled to a personal computer with the PSTrace software (version 4.7) for data acquisition. A platinum plate counter electrode, an Ag/AgCl (3.0 mol L^−1^ KCl) reference electrode and a modified or an unmodified carbon paste working electrode were accommodated in a 15.0 mL conventional three-electrode cell. All experiments were carried out at room temperature (25 ± 2 °C). CV experiments were carried out in 0.4 mmol L^−1^ E2 or E3. A concentration of 1.0 µmol L^−1^ E2 (or E3) was employed to optimize the SWV parameters. The SWV parameters of scan increment (Δ*Es*), amplitude (*a*) and frequency (*f*) were optimized employing a Box-Behnken factorial design using the Statistica software (version 13.3). Box-Behnken design is an experimental design for response surface methodology, which can provide the optimal combination of the operating parameters of SWV for a sensitive determination of estrogens. In this study, the SWV parameters were set as independent variables in Box-Behnken designs and the current intensities were set as responses. The response surfaces generated in the optimization highlighted the best experimental conditions and the behavior of the parameters on the system. Thus, the critical point (maximum point) within the experimental domain studied given to the method high sensitivity. The values that provided the best compromise between the sensitivity and the voltammogram profile were: *∆Es* = 5.4 and 8.0 mV, *a* = 60.0 and 83.0 mV, and *f* = 72.0 and 100.0 Hz for E2 (Table S1; Fig. S1) and E3 (Table S2; Fig. S2), respectively.

In order to evaluate the results provided by the Fe_3_O_4_ NPs-BMI.PF_6_/CPE, UV/VIS spectrometry was used as a comparative technique. The maximum absorbance was observed at 282 nm for E2 and E3, in the concentration ranges of 9.0–216.7 µmol L^−1^ and 10.0–250.0 µmol L^−1^ for E2 and E3, respectively. The spectra were obtained on a UV-1800 spectrophotometer (Shimadzu, Japan) using a quartz cell with an optical path length of 1.0 cm.

## Results and Discussion

### Characterization of the materials

**Figure 1:**
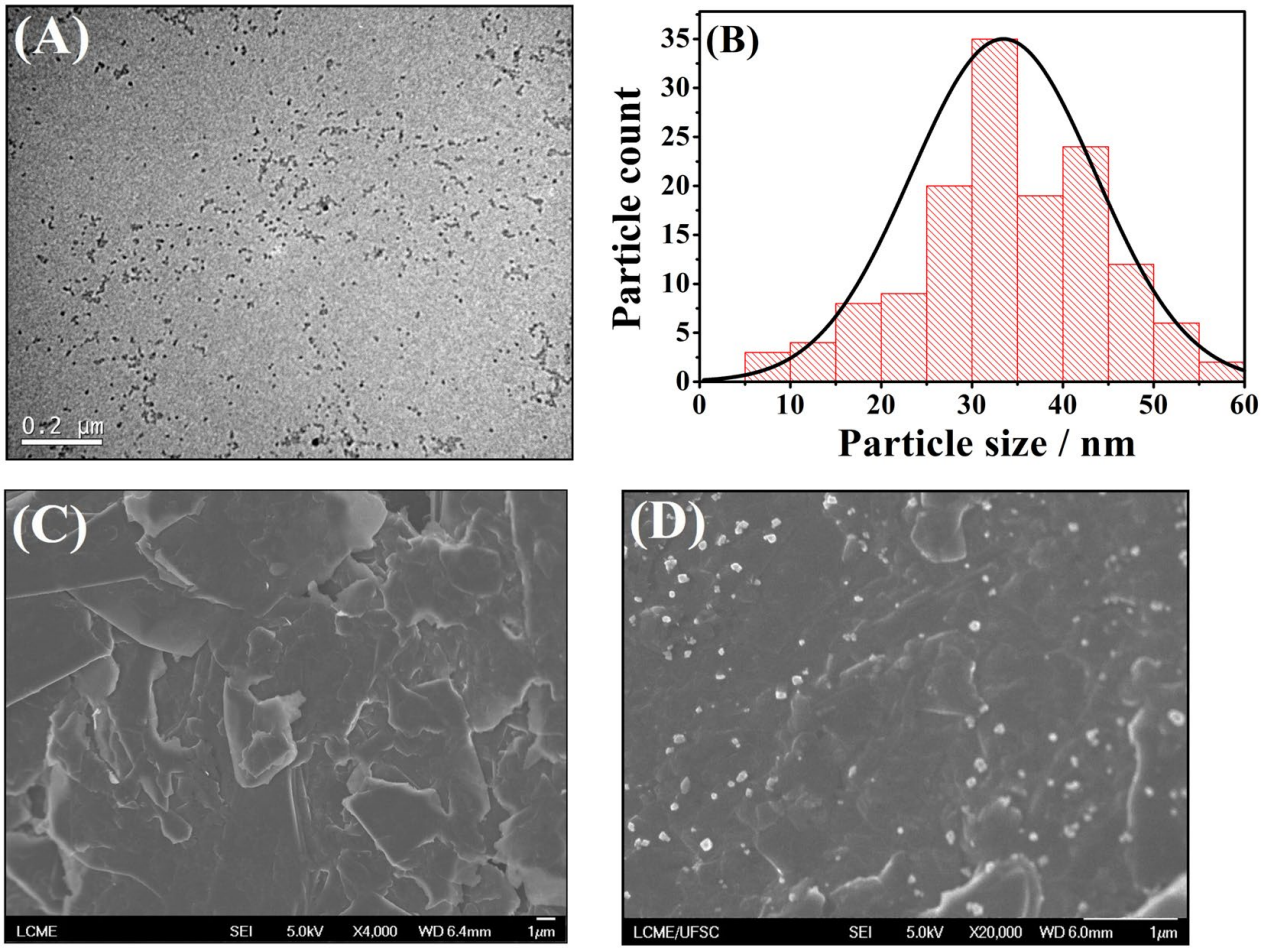
(**A**) TEM image and (**B**) particle size distribution of Fe_3_O_4_ NPs. SEM-FEG images for (**C**) unmodified CPE and (**D**) Fe_3_O_4_ NPs-BMI.PF_6_/CPE.

The characterization of the Fe_3_O_4_ NPs was performed by TEM (Fig. 1A). The image shows the nanoparticles well dispersed in the ionic liquid. A histogram of the particle size distribution (Fig. 1B) was constructed by counting approximately 150 particles randomly chosen from more than one TEM image. The average size estimated of the Fe_3_O_4_ NPs was 33 nm, value not exceeding the limit certified by the manufacturer (<50 nm). The difference between the carbon paste surfaces modified or not was studied by FEG-SEM. The irregular carbon flakes can be observed in the image of CPE (Fig. 1C). However, the SEM-FEG recorded for the Fe_3_O_4_ NPs-BMI.PF_6_/CPE (Fig. 1D) shows the formation of a material with gel aspect, which is associated with the BMI.PF_6_ presence^23^. The Fe_3_O_4_ NPs appear dispersed in this material.

In order to better characterize the surface potential of Fe_3_O_4_ NPs-BMI.PF_6_/CPE, the zeta potential (ζ) was evaluated. The ζ-potential is a measure of the electric charge at the surface of the particles, indicating the physical stability of colloidal systems. As the ζ- potential increases in both the positive (ζ > 30 mV) and negative scale (ζ < −30 mV), the stability of the nanostructures increases^24^. The zeta potentials of −13.2 ± 0.2 mV and +67.2 ± 0.4 mV were obtained for Fe_3_O_4_ NPs and Fe_3_O_4_ NPs-BMI.PF_6_, respectively. The surface potential became positive after modification with BMI.PF_6_, which is related of the positive charge localized in the imidazole ring of ionic liquid^23^. In addition, according to the literature, the Fe_3_O_4_ NPs-BMI.PF_6_ dispersion is considered stable for having a potential zeta value above +30 mV.

### Electrochemical behavior of estrogens at Fe_3_O_4_ NPs-BMI.PF_6_/CPE

**Figure 2:**
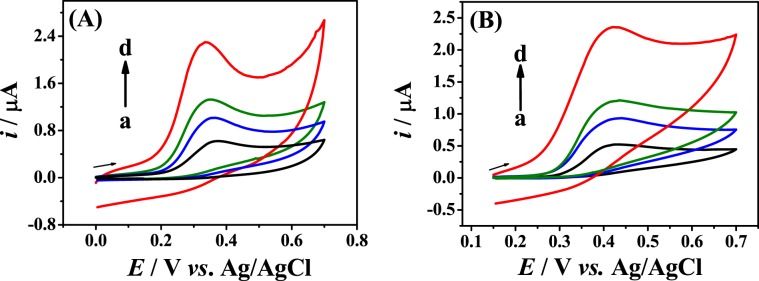
Cyclic voltammograms in the presence of 0.4 mmol L^−1^ E2 (**A**) and E3 (**B**) in 0.2 mol L^−1^ B-R buffer (pH 12.0) at the unmodified CPE (a), Fe_3_O_4_ NPs/CPE (b), BMI.PF_6_/CPE (c) and Fe_3_O_4_ NPs-BMI.PF_6_/CPE (d), *υ* = 50.0 mV s^−1^.

Figure 2 depicts the electrochemical behavior of the estrogens E2 and E3 at different electrodes. In Fig. 2A, the cyclic voltammograms for 0.4 mmol L^−1^ E2 in 0.2 mol L^−1^ B-R buffer (pH 12.0) obtained using the Fe_3_O_4_ NPs-BMI.PF_6_/CPE (d) are shown in comparison with the corresponding results obtained with the electrodes BMI.PF_6_/CPE (c), Fe_3_O_4_ NPs/CPE (b) and the unmodified CPE (a). At the bare CPE (Fig. 2A – curve a), the oxidation peak for E2 was observed at +0.355 V with a current of around 0.42 ± 0.08 μA (*n* = 3). For the Fe_3_O_4_ NPs/CPE (Fig. 2A – curve b) and BMI.PF_6_/CPE (Fig. 2A – curve c), a small increase in the current peak was observed for 0.55 ± 0.04 μA and 0.67 ± 0.07 μA, respectively. In the case of the Fe_3_O_4_ NPs-BMI.PF_6_/CPE (Fig. 2A– curve d), the oxidation peak shifted to +0.320 V and the current for the E2 oxidation was three times higher (1.30 ± 0.07 μA) compared to the bare CPE. The better responses were attributed to the presence of the Fe_3_O_4_ NPs, which provide an increase in the electroactive area of the electrode due to the high porosity of the nanomaterial and the good conductivity provided by BMI.PF_6_^25^. Thus, the use of Fe_3_O_4_ NPs supported in BMI.PF_6_ improves the electrochemical performance of CPE in terms of current intensity (reaction rate). In addition, the peak potential shifted by 35 mV to less positive potentials compared to the unmodified CPE, showing that the electrode modification facilitated the reaction electron transfer. As consequence, the reaction was kinetically and thermodynamically favored on the surface of the modified electrode^26^.

In Fig. 2B, it is clear that E3 displays similar electrochemical behavior as E2, because of the similarity of their chemical structures. Thus, higher peak currents were obtain for E3 using Fe_3_O_4_ NPs-BMI.PF_6_/CPE (Fig. 2B – curve d) and the intensity of 1.06 ± 0.07 μA (*n = *3) at +0.40 V was three times higher compared to the unmodified CPE (0.35 ± 0.02 μA at +0.42 V) (Fig. 2B – curve a). Due to its better electrochemical activity, E2 was selected to represent the estrogens in the experiments to optimize the following parameters: (i) solution pH, (ii) chemical composition of supporting electrolyte and (iii) potential scan rate. Furthermore, the Fe_3_O_4_ NPs-BMI.PF_6_/CPE was applied for the quantification of E2 and E3 in pharmaceutical samples. The results obtained were compared with those provided by the UV/VIS spectrometry.

### Optimization of experimental conditions

**Figure 3:**
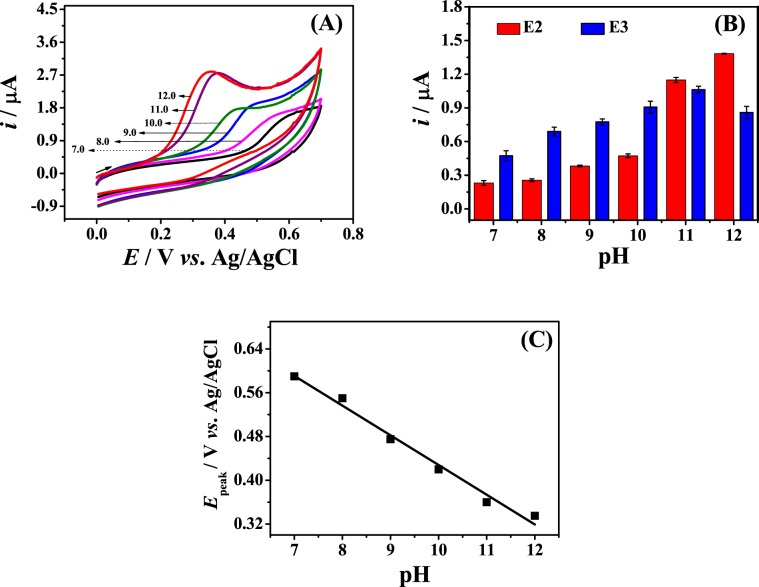
(**A**) Cyclic voltammograms at different pH values a–f (7.0–12.0), (**B**) anodic current and (*n* = 3), (**C**) oxidation potential as a function of solution pH for 0.4 mmol L^−1^ E2 in 0.2 mol L^−1^ B-R buffer at Fe_3_O_4_ NPs-BMI.PF_6_/CPE; *υ* = 50.0 mV s^−1^.

The effect of the solution pH on the electrochemical response to 0.4 mmol L^−1^ E2 at the Fe_3_O_4_ NPs-BMI.PF_6_/CPE was investigated by CV in 0.2 mol L^−1^ B-R buffer solution. Figure 3A shows the cyclic voltammograms obtained under different pH (7.0–12.0) conditions. At pH values lower than 7.0, no redox signal was observed (data not shown) for both E2 and E3. The absence of redox signal of estrogens in acidic medium may be related to poor estrogens interaction with the electrode surface, which leads to low sensitivity of modified electrode under these conditions. As reported in the zeta potential study, the modified electrode surface is positively charged. Thus, an electrostatic interaction between the electrode surface and the estrogens favors the increase of current intensities. This condition is reached in alkaline pHs close or higher than the pKa of estrogens (10.7 for E2 and 10.4 for E3) when they are deprotonated^27^. Thus, the highest current intensities were obtained at pH 12.0 for E2 and pH 11.0 for E3 (Fig. 3B). The oxidation peak potential (*E*_peak_) shifted linearly to less positive potentials as the solution pH increased from 7.0 to 12.0 (Fig. 3C). The linear relation between *E*_peak_ and pH can be expressed as: *E*_peak_ (V) = −0.054 pH + 0.971 (*r* = 0.982). The slope of −0.054 V pH^−1^ is near to the theoretical value of −0.059 V pH^−1^, which denotes that the oxidation of E2 involves the transfer of the same number of protons and electrons. This corroborated with previously reported mechanism for E2 oxidation^7^.

Posteriorly, pH values of 12.0 and 11.0 were selected to investigate the influence of the chemical composition of the supporting electrolyte on the electrochemical oxidation of E2 and E3, respectively. The electrolytes tested were B-R and Ringer’s buffers and NaOH solution, all at a concentration of 0.2 mol L^−1^ (data not shown). The best results for the estrogens E2 and E3 were obtained in B-R buffer. Hence, this buffer was chosen for the subsequent analytical experiments.

### Influence of scan rate

**Figure 4:**
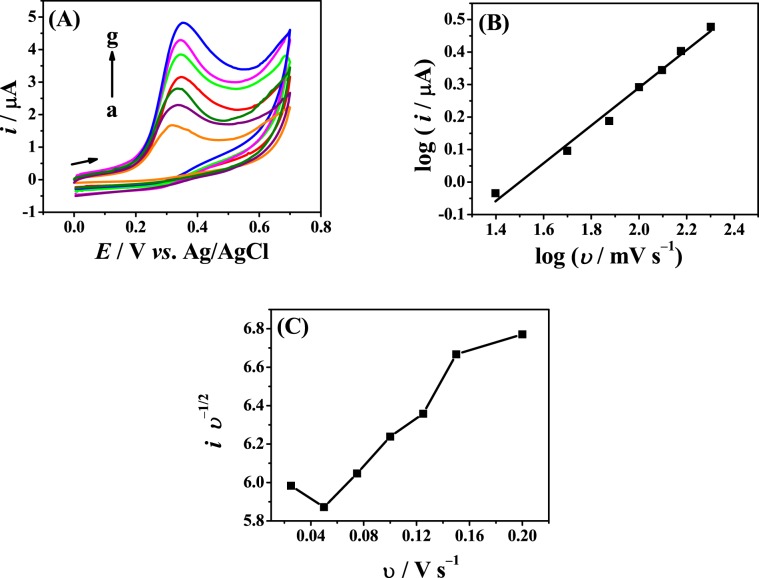
(**A**) Cyclic voltammograms obtained with the Fe_3_O_4_ NPs-BMI.PF_6_/CPE for 0.4 mmol L^−1^ E2 in 0.2 mol L^−1^ B-R buffer (pH 12.0), (a–g) *υ* = 25.0 to 200.0 mV s^−1^, (**B**) plot log *i vs*. log *υ* and (**C**) plot *i υ*^−1/2^ *vs*. *υ*.

The relationship between the scan rate (*υ*) and the oxidation peak current (*i*) for E2 was studied in 0.2 mol L^−1^ B-R buffer (pH 12.0). The oxidation peak current for 0.4 mmol L^−1^ E2 at the Fe_3_O_4_ NPs-BMI.PF_6_/CPE increased with an increase in scan rate in the range of 25.0 to 200.0 mV s^−1^ (Fig. 4A). The peak potential shifted to more positive values with an increase in the scan rate, which is typical of irreversible reactions^28^. The oxidation peak current increased linearly with the square root of the scan rate (*υ*^1/2^) (data not shown), indicating that the electrochemical reaction is diffusion-controlled. The slope of the plot log *i* versus log *υ* (Fig. 4B) was close to 0.5, which confirms that the redox process is mainly controlled by diffusion, with a small contribution from adsorption^28^. The linear regression equation obtained was log *i* = 0.581 log *υ* − 0.871 (*r* = 0.986). In addition, the presence of chemical reactions coupled to the oxidation reaction of E2 was verified through the graph of the current function (*i υ*^−1/2^) versus *υ* (Fig. 4C).

**Figure 5:**
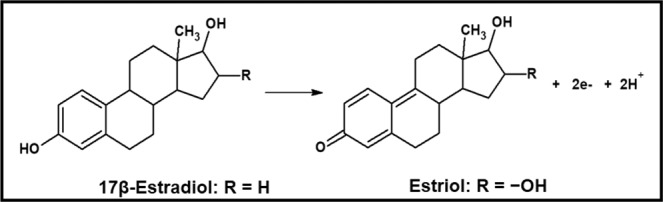
Proposed reaction for 17β-estradiol and estriol oxidation at the Fe_3_O_4_ NPs-BMI.PF_6_/CPE.

Based on the results discussed above, the electrochemical oxidation of E2 mainly produces quinone through an irreversible electrochemical reaction involving the transfer of 2 moles of protons and 2 moles of electrons, with the presence of coupled chemical reactions, as reported in other studies in the literature. The same behavior was verified for E3^7,10,21^. Figure 5 shows the proposed reaction for E2 and E3 oxidation at Fe_3_O_4_ NPs-BMI.PF_6_/CPE.

### Calibration plots

**Figure 6:**
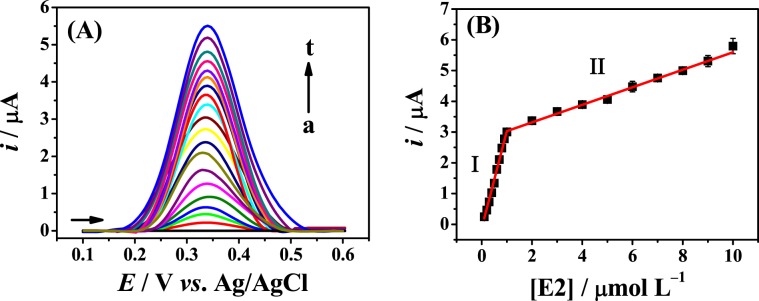
(**A**) Square-wave voltammograms for the blank (a) and different concentrations of E2 (0.1–10.0 μmol L^−1^) (b–t) in 0.2 mol L^−1^ B-R buffer solution (pH 12.0) at the Fe_3_O_4_ NPs-BMI.PF_6_/CPE, Δ*Es* = 5.4 mV, *a* = 60.0 mV, and *f* = 72.0 Hz and (**B**) calibration plot for E2 (*n* = 8).

Applying the optimized conditions, the calibration plots for E2 and E3 (*n* = 8) were constructed by SWV, using the proposed modified electrode. The square-wave voltammograms obtained for different concentrations of E2 are shown in Fig. 6A. Well-defined peaks can be observed for E2 at +0.340 V and E3 at +0.350 V (Fig. S3A). The calibration plot for E2 (Fig. 6B) displays two ranges of linearity: the first (I) from 0.10 to 1.0 μmol L^−1^: *i*/μA = 3.24 (±0.08) [E2]/μmol L^−1^ −0.19 (±0.05) (*r* = 0.995) and the second (II) from 1.0 to 10.0 μmol L^−1^: *i*/μA = 0.29 (±0.01) [E2]/μmol L^−1^ + 2.72 (±0.05) (*r* = 0.993). In the case of the calibration plot for E3 (Fig. S3B) the linear intervals were: (I) from 1.0 to 10.0 μmol L^−1^: *i*/μA = 0.10 (±4.0 × 10^−3^) [E3]/μmol L^−1^ −0.07 (±0.01) (*r* = 0.997) and (II) from 10.0 to 110.0 μmol L^−1^: *i*/μA = 0.03 (±7.0 × 10^−4^) [E3]/μmol L^−1^ + 0.97 ±0.05) (*r* = 0.997). The two linear ranges with distinct slopes may be related to the saturation of the electrode surface. With an increase in E2 or E3 concentration, the interaction of the molecules with the surface of the modified electrode decreases, which explains the reduction in sensitivity^29^.

The limit of detection (LOD) and limit of quantification (LOQ) were calculated according to the equations: LOD = 3 × Sb/B and LOQ = 10 × Sb/B, where Sb is the standard deviation of the linear coefficient and B is the slope of the calibration plot. Using the linear regression with higher sensitivity, the LOD values obtained for E2 and E3 were 0.05 and 0.30 μmol L^−1^ respectively; and the LOQ values for E2 and E3 were 0.15 and 1.00 μmol L^−1^, respectively.

For comparison purposes, the calibration plots for E2 and E3 were constructed using the bare CPE, under the same experimental conditions (data not shown). For E2, the linear range was from 0.9 to 10.0 μmol L^−1^ (*r* = 0.998) with a linear regression of *i*/µA = 0.23 (±2.0 × 10^−4^) [E2]/μmol L^−1^ + 0.04 (±0.03). For E3 the linear range was from 20.0 to 180.0 µmol L^−1^ (*r* = 0.997) with a linear regression of *i*/µA = 0.01 (±1.0 × 10^−3^) [E3]/μmol L^−1^ −0.07 (±0.03). The LOD and LOQ values obtained for E2 were 0.4 and 1.3 μmol L^−1^, respectively, and for E3 the corresponding values were LOD = 9.0 μmol L^−1^ and LOQ = 30.0 μmol L^−1^. These results indicate that the Fe_3_O_4_ NPs-BMI.PF_6_/CPE is more sensitive for E2 and E3 analysis than the bare CPE. As showed, the analytical sensitivity (slope of the calibration plot) was 0.23 and 0.01 µA L μmol^−1^ for E2 and E3, respectively at GCE. On Fe_3_O_4_ NPs-BMI.PF_6_/CPE, the sensitivity increased to 3.24 and 0.10 µA L μmol^−1^ for E2 and E3, respectively. Both the bare CPE and the Fe_3_O_4_ NPs-BMI.PF_6_/CPE were more sensitives for detecting E2 than for E3. This result can be explained provided the interaction of the estrogens with the electrode surface does not just depend on physico-chemical factors related to the energy involved in the interaction processes. The interaction is also dependent on stereochemical factors, which can establish limitations regarding the diffusivity of an estrogen in a porous electrode material and impose difficulties to reach interaction with active sites, for example. Thus, even if different estrogens obtain to current peaks at similar redox potential, is possible that these distinct estrogens obtain to different sensitivities for the same electrode. As in this work, other published studies on estrogens report a better electrochemical activity of E2 compared to E3, which may explain a better sensitivity for E2 detection^30–33^.

Table 1 lists published papers reporting studies on E2 and E3 analysis. It can be observed that the LODs obtained with the Fe_3_O_4_ NPs-BMI.PF_6_/CPE are within the range of detection limits reported in the recent literature. It should also be noted that besides being able to detect at the nanomolar level, as in the case of other instrumental methods of analysis, the electroanalytical techniques offer portability of the equipment, low maintenance cost, and ease of preparation and operation of the electrodes.

**Table 1:** Performance of different methods for E2 and E3 detection.

Technique	LOD/nmol L^−1^		Reference
	E2	E3	
FIA-EC^a^	100.0	100.0	^34^
HPLC/UV	1.0	0.8	^35^
HPLC/MS	0.7	0.3	^36^
CE^b^	220.0	138.7	^37^
**Voltammetric technique**			
rGO/CuTthP^c^/GCE	5.3	—	^1^
Fe_3_O_4_ NPs-MIP^d^/SPCE	20.0	—	^2^
CuO^e^/CPE	21.0	—	^7^
Fe_3_O_4_ NPs/CPE	—	2750.0	^10^
CNTs^f^/SPCE	—	530.0	^16^
CNB-Ag NPs^g^/GCE	—	160.0	^11^
Pt-MWCNTs^h^/GCE	180.0	620.0	^33^
Fe_3_O_4_ NPs-BMI.PF_6_/CPE	50.0	300.0	This work

^a^FIA-EC – flow injection analysis with amperometric detection;

^b^CE – capillary electrophoresis;

^c^rGO/CuTthP – reduced graphene oxide with Cu(II) porphyrin complex;

^d^MIP – molecularly imprinted polymer;

^e^CuO – copper(II) oxide;

^f^CNTs – carbon nanotubes;

^g^CNB-Ag NPs – carbon black nanoballs decorated with silver nanoparticles;

^h^Pt-MWCNTs – platinum nano clusters electrodeposited on multi-walled carbon nanotubes.

### Repeatability and interference studies with the Fe_3_O_4_ NPs-BMI.PF_6_/CPE

The intra-day repeatability provided by the Fe_3_O_4_ NPs-BMI.PF_6_/CPE was assessed by eight measurements of peak currents in the same day using the same electrode, the surface being renewed after each measurement. The measurements were carried out at four different estrogen concentrations in 0.2 mol L^−1^ B-R buffer solution: 0.3, 1.0, 5.0 and 10.0 μmol L^−1^ for E2 (pH 12.0) and 5.0, 10.0, 60.0 and 110.0 μmol L^−1^ for E3 (pH 11.0). The relative standard deviation (*n* = 8) varied from 3.4% for the highest concentration to 3.8% for the lowest concentration for E2 and for E3 it was lower than 3.2%. To assess the inter-day repeatability, a single sensor was used, and the same procedure applied for the measurements was repeated on eight consecutive days. The relative standard deviation (*n* = 8) varied from 2.2% for the highest concentration to 4.7% for the lowest concentration. These results demonstrate the excellent repeatability furnished by the Fe_3_O_4_ NPs-BMI.PF_6_/CPE when applied for the detection of E2 and E3.

In order to evaluate the selectivity of the Fe_3_O_4_ NPs-BMI.PF_6_/CPE toward E2 and E3 in the presence of other chemical species, the influence of potential interfering substances on the E2 and E3 anodic currents was investigated. The excipients present in the pharmaceutical preparation used in this study (i.e., amido, lactose, magnesium stearate, sodium edetate, propylene glycol, carbopol 990, sodium hydroxide, glycerol, stearic acid, lanette and sorbitol) were tested as potential interferents. The E2/interferent and E3/interferent ratios tested were 1/0.01, 1/0.1, 1/1, 1/10 and 1/100. The peak current related to the oxidation of E2 and E3 did not change significantly even in the presence these potential interferents, with a coefficient of variation lower than 5.0%. These results showed that the Fe_3_O_4_ NPs-BMI.PF_6_/CPE can be used for the determination of E2 and E3 in the presence of these compounds without any significant matrix interference effects. However, a simultaneous determination of E2 and E3 was not possible with Fe_3_O_4_ NPs-BMI.PF_6_/CPE, since the estrogen oxidation peaks are at close potentials (+0.340 V for E2 and +0.330 V for E3). In that regard, it would be possible to accomplish a determination of total estrogens.

### Determination of estrogens in pharmaceutical samples

**Figure 7:**
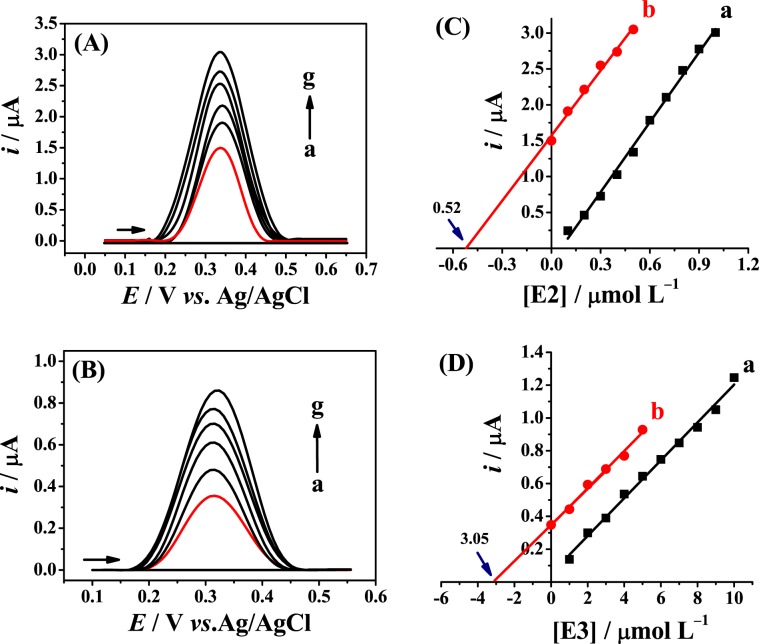
(**A**) Square-wave voltammograms for E2 in the tablet sample: blank (a), sample (b) and sample with successive additions (c–g) of E2 standard solution in 0.2 mol L^−1^ B-R buffer (pH 12.0) obtained at the Fe_3_O_4_ NPs-BMI.PF_6_/CPE, Δ*Es* = 5.4 mV, *f = *72.0 Hz and *a* = 60.0 mV. (**B**) Square-wave voltammograms for E3 in tablet sample: blank (a), sample (b), and sample with successive additions (c–g) of E3 standard solution in 0.2 mol L^–1^ B-R buffer (pH 11.0) obtained at the Fe_3_O_4_ NPs-BMI.PF_6_/CPE, Δ*Es* = 8.0 mV, *f* = 100.0 Hz and *a* = 83.0 mV. (**C**,**D**) Calibration plot (a) and standard addition plot (b) for E2 and E3, respectively.

The Fe_3_O_4_ NPs-BMI.PF_6_/CPE prepared under optimized conditions was used for the quantification of E2 and E3 in pharmaceutical samples prepared as described in the Experimental section. The electroanalytical determination of E2 was performed with tablet (Fig. 7A) and gel (Fig. S4) samples and E3 was determined in tablet (Fig. 7B) and cream (Fig. S5) samples. The determinations were carried out by the standard addition method, with eight replicates and without prior sample treatment. The square-wave voltammograms for E2 and E3 in the tablet samples are shown in Fig. 7A,B, respectively. Figure 7C,D show the calibration plots (curve a) and the standard addition plots (curve b) for E2 and E3 in the tablet samples, respectively. The linear regressions were obtained for the corresponding standard addition plots for the samples C (*i*/µA = 3.02[E2]/μmol L^−1^ + 1.57) and D (*i*/µA = 0.11[E3]/μmol L^−1^ + 0.34). It can be observed, for both samples, that the slopes of the standard addition plot and the calibration plot are quite similar, confirming the matrix components did not affect the determination of E2 and E3. Thus, these results emphasize that the E2 and E3 concentrations can be quantified with high sensitivity and selectivity using the modified electrode.

The accuracy provided by the Fe_3_O_4_ NPs-BMI.PF_6_/CPE for the determination of E2 and E3 was evaluated by comparison with the data furnished by the UV/VIS technique. Table 2 summarizes the results obtained for E2 in the tablet and gel samples. The relative standard deviation (RSD) values obtained for the two samples were 3.85% measured with the modified electrode and 2.16 and 2.59% using the UV/VIS technique. The relative errors between the modified electrode and the UV/VIS technique were +0.97 and +1.96%, for the tablets and gel samples, respectively. The t-test and F-test applied to this dataset demonstrated a confidence level of 95% for the reliability of the data acquired with the Fe_3_O_4_ NPs-BMI.PF_6_/CPE and the UV/VIS technique.

**Table 2:** Determination of E2 in pharmaceutical samples in tablet and gel forms.

Sample Technique	Tablets		Gel	
	UV/VIS	SWV	UV/VIS	SWV
Sample (mg)	1.00	1.00	0.50	0.50
Found value (mg)^a^	1.03	1.04	0.51	0.52
RSD (%)	2.59	3.85	2.16	3.85
E_r_ (%)^b^	+0.97	+1.96		
t_value_^c^	0.71	1.41		
F_value_^d^	2.24	3.30		

^a^*n* = 8;

^b^E_r_ = Relative error between SWV results with the proposed Fe_3_O_4_ NPs-BMI.PF_6_/CPE and UV/VIS spectrometry;

^c^t_critical_ = 2.36;

^d^F_critical_ = 3.79.

Additional results, including the values determined for E3 using the spectroscopic method and statistical data, are shown in Table 3. Similarly, outstanding results were obtained in the determination of E3 in tablets and cream samples. This set of experiments confirmed that the Fe_3_O_4_ NPs-BMI.PF_6_/CPE supplied accurate and precise data for the determination of E2 and E3 in pharmaceutical samples.

**Table 3:** Determination of E3 in pharmaceutical samples in tablet and cream form.

Sample Technique	Tablets		Cream	
	UV/VIS	SWV	UV/VIS	SWV
Sample (mg)	1.00	1.00	1.00	1.00
Found value (mg)^a^	1.02	1.01	1.02	1.04
RSD (%)	1.96	2.08	2.94	3.85
E_r_ (%)^b^	−0.98		+1.96	
t_value_^c^	1.35		1.41	
F_value_^d^	1.10		1.78	

^a^*n* = 8;

^b^E_r_ = Relative error between SWV results with the proposed Fe_3_O_4_ NPs-BMI.PF_6_/CPE and UV/VIS spectrometry;

^c^t_critical_ = 2.36;

^d^F_critical_ = 3.79.

## Conclusions

The Fe_3_O_4_ NPs-BMI.PF_6_/CPE was used together SWV and successfully applied for the individual determination of E2 and E3 in pharmaceutical samples. The experimental conditions of SWV were optimized using Box-Behnken factorial design for each estrogen ensuring greater sensitivity in the analysis. The modified electrode is a low-cost assembly; the surface is easily renewable and in association with SWV, it provided low detection and quantification limits, as well as high sensibility and excellent intra- and inter-day data repeatability. However, a simultaneous determination of E2 and E3 was not possible, since the oxidation peaks are in similar potentials. Notwithstanding, it was observed the absence of interference in the individual determination of these hormones in complex samples. The results were compared to those provided by UV/VIS spectrometry in terms of accuracy and precision of data provided. The statistical tests demonstrated the reliability of the data acquired with the Fe_3_O_4_ NPs-BMI.PF_6_/CPE and the UV/VIS technique. Hence, additional benefits of Fe_3_O_4_ NPs-BMI.PF_6_/CPE should be noted: high accuracy and precision provide safety in data evaluation, it is suited for quantitative analysis of organics molecules which can be oxidized on the electrode, and the matrices components do not interfere in the analysis. Obviously, the electroanalytical instrumentation is rather inexpensive compared with spectrometric methods and can be made so small and lightweight that it can be used in field analysis by portable instruments with batteries. Finally, the findings allow to conclude that the proposed Fe_3_O_4_ NPs-BMI.PF_6_/CPE, is a promiser modified electrode for use in quality control of E2 and E3 estrogens in pharmaceutical industry.

## Supplementary information

Supplementary Information.
