# Supplementary material for: Ionic liquid-supported magnetite nanoparticles as electrode modifier materials for estrogens sensing
Source: Sci Rep. 2020 Feb 6;10:1955. doi: 10.1038/s41598-020-58931-6 (PMC7005039; doi:10.1038/s41598-020-58931-6)
Supplement: Supplementary file 1 — Supplementary Information. [file 41598_2020_58931_MOESM1_ESM.doc]

**Supplementary Material**

Ionic liquid-supported magnetite nanoparticles as electrode

modifier materials for estrogens sensing

Fernanda Moreira, Edson Roberto Santana, Almir Spinelli*

*Grupo de Estudos de Processos Eletroquímicos e Eletroanalíticos*

*Universidade Federal de Santa Catarina*

*Campus Universitário Reitor João David Ferreira Lima*

*Departamento de Química* – *CFM*

*88040-900* – *Florianópolis, SC, Brazil*

*Corresponding author: Tel.: +55-48-37219778

*E-mail address*: almir.spinelli@ufsc.br

**Table S1.** Optimization of the parameters of the square wave voltammetry technique for E2.

| Assay | Frequency | Amplitude | Increment | *i* (µA) |
| --- | --- | --- | --- | --- |
| 1 | -1 (30) | -1 (20) | 0 (5) | 0.17 |
| 2 | 1 (100) | -1 (20) | 0 (5) | 0.50 |
| 3 | -1 (30) | 1 (90) | 0 (5) | 1.48 |
| 4 | 1 (100) | 1 (90) | 0 (5) | 0.49 |
| 5 | -1 (30) | 0 (55) | -1 (2) | 1.10 |
| 6 | 1 (100) | 0 (55) | -1 (2) | 1.13 |
| 7 | -1 (30) | 0 (55) | 1 (8) | 0.83 |
| 8 | 1 (100) | 0 (55) | 1 (8) | 2.46 |
| 9 | 0 (65) | -1 (20) | -1 (2) | 0.68 |
| 10 | 0 (65) | 1 (90) | -1 (2) | 2.15 |
| 11 | 0 (65) | -1 (20) | 1 (8) | 1.25 |
| 12 | 0 (65) | 1 (90) | 1 (8) | 1.70 |
| 13 | 0 (65) | 0 (55) | 0 (5) | 2.89 |
| 14 | 0 (65) | 0 (55) | 0 (5) | 2.58 |
| 15 | 0 (65) | 0 (55) | 0 (5) | 2.57 |
| 16 | 0 (65) | 0 (55) | 0 (5) | 2.74 |
| 17 | 0 (65) | 0 (55) | 0 (5) | 2.98 |

**Table S2.** Optimization of the parameters of the square wave voltammetry technique for E3.

| Assay | Frequency | Amplitude | Increment | *i* (µA) |
| --- | --- | --- | --- | --- |
| 1 | -1 (40) | -1 (30) | 0 (5) | 0.90 |
| 2 | 1 (100) | -1 (30) | 0 (5) | 1.03 |
| 3 | -1 (40) | 1 (90) | 0 (5) | 0.73 |
| 4 | 1 (100) | 1 (90) | 0 (5) | 2.27 |
| 5 | -1 (40) | 0 (60) | -1 (2) | 0.96 |
| 6 | 1 (100) | 0 (60) | -1 (2) | 1.73 |
| 7 | -1 (40) | 0 (60) | 1 (8) | 0.80 |
| 8 | 1 (100) | 0 (60) | 1 (8) | 2.86 |
| 9 | 0 (70) | -1 (30) | -1 (2) | 1.43 |
| 10 | 0 (70) | 1 (90) | -1 (2) | 1.08 |
| 11 | 0 (70) | -1 (30) | 1 (8) | 1.86 |
| 12 | 0 (70) | 1 (90) | 1 (8) | 1.71 |
| 13 | 0 (70) | 0 (60) | 0 (5) | 2.30 |
| 14 | 0 (70) | 0 (60) | 0 (5) | 2.36 |
| 15 | 0 (70) | 0 (60) | 0 (5) | 2.17 |
| 16 | 0 (70) | 0 (60) | 0 (5) | 2.06 |
| 17 | 0 (70) | 0 (60) | 0 (5) | 2.04 |


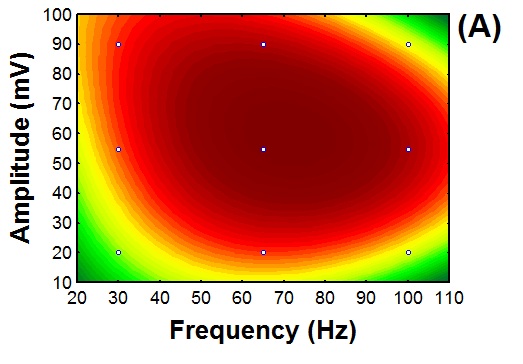

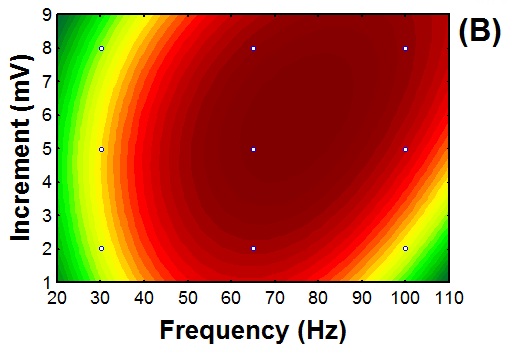


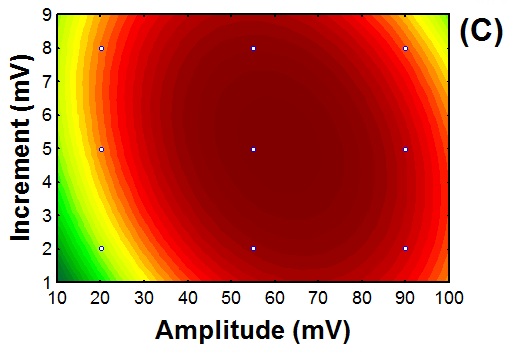


**Fig. S1.** Response surface for E2.


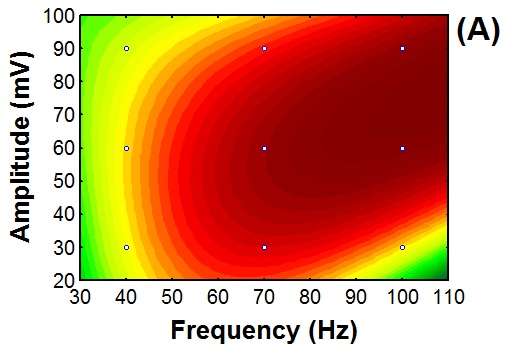

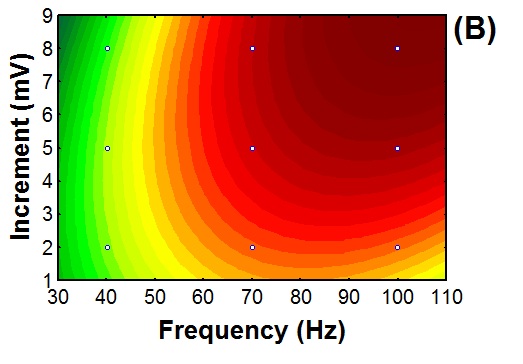


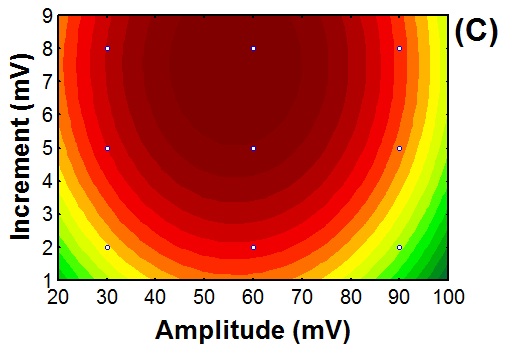


Fig. S2. Response surface for E3.

**Fig. S3.** (A) Square-wave voltammograms for the blank (a) and different concentrations of E3 (1.0–110.0 μmol L^−1^) (b–u) in 0.2 mol L^−1^ B-R buffer solution (pH 11.0) at the Fe_3_O_4_ NPs-BMI.PF_6_/CPE; Δ*Es* = 8.0 mV, *a* = 83.0 mV, and *f* = 100.0 Hz. (B) Calibration plot for E3 (*n* = 8).

**Fig. S4.** (A) Square-wave voltammograms for the sample in gel of E2: blank (a), sample (b), sample with successive additions (c–g) of E2 standard solution in 0.2 mol L^–1^ B-R buffer (pH 12.0) obtained at the Fe_3_O_4_ NPs-BMI.PF_6_/CPE; Δ*Es* = 5.4 mV, *f* = 72.0 Hz and *a* = 60.0 mV. (B) calibration plot (a) and standard addition plot (b).

**Fig. S5.** (A) Square-wave voltammograms for the sample in cream of E3: blank (a), sample (b), sample with successive additions (c–g) of E3 standard solution in 0.2 mol L^–1^ B-R buffer (pH 11.0) obtained at the Fe_3_O_4_ NPs-BMI.PF_6_/CPE, Δ*Es* = 8.0 mV, *f* = 100.0 Hz and *a* = 83.0 mV. (B) calibration plot (a) and standard addition plot (b).
